# Ensemble Dilated Convolutional Neural Network and Its Application in Rotating Machinery Fault Diagnosis

**DOI:** 10.1155/2022/6316140

**Published:** 2022-09-21

**Authors:** Yuxiang Cai, Zhenya Wang, Ligang Yao, Tangxin Lin, Jun Zhang

**Affiliations:** School of Mechanical Engineering and Automation, Fuzhou University, Fuzhou 350108, China

## Abstract

Fault diagnosis of rotating machinery is an attractive yet challenging task. This paper presents a novel intelligent fault diagnosis scheme for rotating machinery based on ensemble dilated convolutional neural networks. The novel fault diagnosis framework employs a model training strategy based on early stopping optimization to ensemble several one-dimensional dilated convolutional neural networks (1D-DCNNs). By varying the dilation rate of the 1D-DCNN, different receptive fields can be obtained to extract different vibration signal features. The early stopping strategy is used as a model update threshold to prevent overfitting and save computational resources. Ensemble learning uses a weighted mechanism to combine the outputs of multiple 1D-DCNN subclassifiers with different dilation rates to obtain the final fault diagnosis. The proposed method outperforms existing state-of-the-art classical machine learning and deep learning methods in simulation studies and diagnostic experiments, demonstrating that it can thoroughly mine fault features in vibration signals. The classification results further show that the EDCNN model can effectively and accurately identify multiple faults and outperform existing fault detection techniques.

## 1. Introduction

Rotating machinery is widely used in manufacturing, transportation, aerospace, and other industries [1, 2]. However, rotating machinery systems frequently operate in high-speed, heavy-duty environments, inevitably resulting in internal components (such as bearings and gears) that are susceptible to damage. While the efficiency of rotating machinery can be reduced by minor failures, the consequences of serious failures can be catastrophic. Furthermore, vibration signals monitored in harsh industrial environments are subject to significant noise interference, which poses a significant challenge for robust fault diagnosis. Fortunately, with the rapid development and integration of sensor technology in the modern industry, condition monitoring and fault diagnosis have become the most effective methods to avoid damage using the measured monitoring vibration signals [3, 4]. As a result, prognostics and health management (PHM) of rotating machinery under changeable working circumstances has emerged as a critical technique for economic efficiency and a hot topic of various research studies [5].

### 1.1. Problems and Motivation

The diagnosis of rotating machinery faults is essentially a pattern recognition issue related to the health condition. Traditional fault diagnosis techniques, such as the wavelet transform [6, 7], variable modal decomposition [8, 9], and empirical modal decomposition [10–13], are challenging to extract fault discriminative features from vibration signals with nonstationary and nonlinear characteristics and demand excessive expertise and expert knowledge, limiting their practical application. Furthermore, the development of artificial intelligence technologies has increased their application in a variety of industries, such as mechanical fault diagnostics. Intelligent fault diagnosis has two main forms: machine learning combined with manual feature extraction [14, 15] or deep learning with automated feature extraction [16–18]. Deep learning-based approaches have gained a lot of attention and popularity as a result of their ability to achieve good end-to-end fault diagnosis and automated fault feature extraction. Traditional fault diagnosis methods or a combination of manual feature extraction and machine learning cannot accomplish the task [19, 20].

While deep learning-based mathematical frames decrease the requirement for expert knowledge and manual feature engineering, it is an effective tool for mechanical fault identification. Artificial neural networks (ANNs), recurrent neural networks (RNNs), and convolutional neural networks (CNNs) are the most common deep learning techniques. For example, Moosavi et al. [21] used a multilayer ANN for fault detection and diagnosis of electric motors. Mao et al. [22] proposed a semirandom subspace method with a bidirectional gate recurrent unit (a modified RNN algorithm) to take full advantage of fusion features for bearing fault diagnosis. Wu and Ma [23] proposed an improved RNN method for wind turbine fault diagnosis based on long short-term memory and Kullback–Leibler divergence. The abovementioned deep learning-based research approaches produced good fault diagnostic conclusions. However, when compared to the other two deep learning approaches, the ANN-based diagnostic method suffers from weak nonlinear fitting ability. Furthermore, the RNN-based diagnostic technique suffers from gradient dispersion and gradient explosion conundrum in model training, as well as containing too many model parameters.

In this work, CNN was chosen over the other approaches because of its superior region feature extraction capabilities and unique model parameter sharing mechanism [24]. Many experts and researchers have conducted extensive research on CNN models. For example, Chen et al. [25] suggested a rolling element-bearing fault approach based on cyclic spectrum consistency and CNN to achieve high diagnostic accuracy. Plakias and Boutalis [26] proposed an attention-intensive CNN with improved generalization capabilities for recognizing rolling element-bearing faults. Guo et al. [27] developed a fault diagnosis model capable of reliable and quick fault identification of multichannel data utilizing multilinear principal component analysis and CNN. Han et al. [28] suggested a CNN-support vector machine system with high robustness in diagnosing bearing faults.

### 1.2. Proposed Methods

However, the abovementioned CNN-based fault diagnosis method achieves advanced diagnostic performance due to its robust local feature extraction and flexible structure. The abovementioned CNN-based fault diagnosis research, on the other hand, has such limitations as follows:

1. The above CNN models are constrained by the classic convolution process, which is incapable of accurately diagnosing faults in complicated industrial diagnostic situations.
2. In the case of a single receptive field (RF), fault diagnosis of the CNN frequently relies on a few feature maps to create unreliable judgments, posing a significant risk to decision-making.

Therefore, the purpose of this study is to investigate a mechanical health monitoring method with strong robustness in order to reduce the negative noise impact under various complex operating situations. To address the aforementioned limitations of classic CNN, this paper proposes an intelligent rotating machinery fault diagnosis model based on the ensemble dilated convolutional neural network (EDCNN) and early stopping optimization. Dilated convolutional neural network (DCNN) not only has a large RF but can also maintain the size of the model. EDCNN takes the concept of ensemble learning and applies it to fault classification by ensembling multiple weak classifiers to jointly consider multiple feature maps for decision making.

### 1.3. Contributions and Structure of This Paper

The main contributions of this work are as follows:

1. A novel deep learning algorithm called EDCNN is proposed, which ensembles multiple dilated convolutional neural networks with different dilation rates to extract features effectively.
2. An intelligent model training approach based on early stopping optimization is implemented. This technique conserves computing resources while minimizing overfitting and performance degradation.
3. A novel EDCNN-based fault diagnosis framework applied to rotating machinery is proposed. The effectiveness and superiority of the proposed method are confirmed by the benchmark rolling bearing dataset and the wind turbine simulator dataset.

The rest of this paper can be summarized as follows. The proposed EDCNN and the suggested EDCNN-based intelligent fault diagnostic method for rotating machinery are described in Section 2. The proposed fault diagnostic model is validated using the rolling bearing and wind turbine datasets in Section 3. Finally, the main conclusions are summarized in Section 4.

## 2. Intelligent Fault Diagnosis Method for Rotating Machinery Based on the EDCNN

In this section, the basic theory of the proposed EDCNN method is first discussed. Subsequently, the proposed framework for intelligent fault diagnosis is presented.

### 2.1. Mathematical Model of the Proposed EDCNN

#### 2.1.1. One-Dimensional Dilated Convolutional Neural Network (1D-DCNN)

Deep learning-based fault diagnosis techniques have attracted widespread attention and have been extensively studied and applied. ANN is a mathematical model that simulates the activity mechanism of the human nervous system by computing the weight of each neuron on all neurons connected layer to layer. When neurons are overstacked, however, the computing resources are too enormous and the capacity to extract features is extremely limited. The RNN can extract temporal information more efficiently than ANN, but its nonparallel computing strategy will give training an appropriate diagnostic model harder. As a result, CNN was chosen by the authors for the research of deep learning in fault diagnosis.

Dilated convolutional neural networks are modified convolutional neural networks that are used for multipattern identification and sensitive feature extraction in complicated tasks. The same model volume can be captured efficiently with a more comprehensive range of RFs. In this work, the time-series signals are fed into a deep learning model, the diagnostic model extracts the characteristics of the input signals adaptively, and the final output is utilized to make the final conclusion.

**Figure 1:**
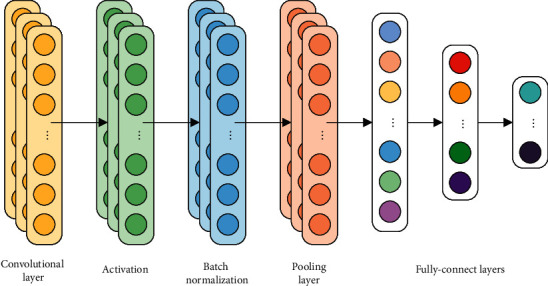
Structure of the one-dimensional convolutional neural network.

**Figure 2:**
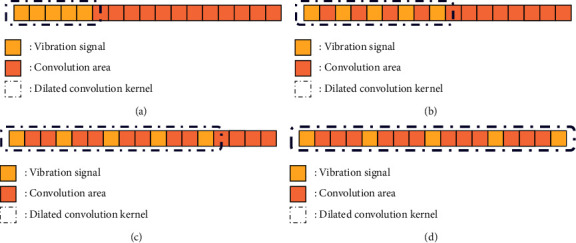
Convolution region with different dilation rates. (a) DR = 1, (b) DR = 2, (c) DR = 3, and (d) DR = 4.

Similar to the CNN model, the DCNN model consists of convolutional layers, pooling layers, activations, batch normalizations, and fully connected layers [29–31] as shown in Figure 1. Convolutional layers could extract features by producing highly focused and continuous information. The dilated convolution kernel (DCK) has a hyperparameter called the dilation rate (DR) that primarily indicates the dilation scale when compared to the normal convolution kernel. With DCK, RF can be dilated to capture different feature components without increasing the size of the convolution kernel. The ensemble model in this study is composed of subclassifiers 1, 2, 3, and 4, which use dilated convolution kernels with dilation rates of 1, 2, 3, and 4, respectively. The following equation expresses the dilation convolution process:

(1) $$\begin{array}{c} C_{j}^{n}=\underset{i\in M_{j}}{\sum }X_{i}^{n-1}∙W_{ij}^{n}+b_{j}^{n}\text{,} \end{array}$$

where *C*_*j*_^*n*^ is the *j* th element of the *n* th convolutional layer, *M*_*j*_ is the convolution region of the input signal, which varies with DR, as shown in Figure 2, *X*_*i*_^*n*−1^ is the previous layer output inside *M*_*j*_, *W*_*ij*_^*n*^ is the weight matrix of the corresponding convolution kernel, and *b*_*j*_^*n*^ is the bias. The activation follows convolutional layers, and the exponential linear unit (ELU) activation function is chosen and denoted as follows:

(2) $$\begin{array}{c} ELU\left(x\right)=\left\{\begin{array}{cc} x, & if\;x>0\text{,}\\ \alpha *\left(\exp\left(x\right)-1\right), & \;if\;x\leq 0^{\prime }\text{,} \end{array}\right.\begin{array}{c} \; \end{array} \end{array}$$

where *x* is the input of neural network model. The activation is a nonlinear function that transforms input values and enhances the ability of the network to express nonlinearity. Lastly, *α* is a hyperparameter taken as 1 in this paper.

Pooling layers are used to accomplish sparse processing while assuring a low number of neurons and comprehensive feature representation. Max pooling, mean pooling, and stochastic pooling are all standard pooling methods. In this paper, the max pooling method is used and calculated as follows:

(3) $$\begin{array}{c} M_{m,n,k}^{l}={max\;pooling}_{\left(m,n\right)\in R_{i,j}^{l}}^{l}\left(x_{m,n,k}^{l-1}\right)\text{,} \end{array}$$

where *M*_*m*,*n*,*k*_^*l*^ is the computed value of location (*i*, *j*) in the *k* th feature map of the *l* th layer after the pooling operation, *R*_*i*,*j*_^*l*^ is the pooling area around the location (*i*, *j*), and *x*_*m*,*n*,*k*_^*l*−1^ is the node at the location (*m*, *n*) in the pooling domain.

Batch normalization is used to normalize the input data into the network model in order to speed up the training process while preserving as much expressiveness as possible. The following is a description of the batch normalization:

(4) $$\begin{array}{c} \begin{array}{c} \mu =\frac{1}{N_{batch}}\overset{N_{batch}}{\underset{s=1}{\sum }}x_{s}, \end{array}\\ \sigma^{2}=\frac{1}{N_{batch}}\overset{N_{batch}}{\underset{s=1}{\sum }}{\left(x_{s}-\mu \right)}^{2}\text{,}\\ {\hat{x}}_{s}=\frac{x_{s}-\mu }{\sqrt{\sigma^{2}+\varepsilon }}\text{,}\\ y_{s}=\gamma \hat{x}+\beta \text{,} \end{array}$$

where *N*_*batch*_ represents the number of small batches of data, *x*_*s*_ represents the *s* th input, *μ* and *σ*^2^ represent the mean and variance of small batches of data, respectively, *ε* represents a constant close to but greater than 0, ${\hat{x}}_{s}$ represents the result of normalizing the data, *γ* and *β* define the parameters that can be learned by the network, and *y*_*s*_ represents the *s* th output of the data after batch normalization.

The fully connected layer performs feature categorization after numerous layered convolutional blocks. It takes place on the utterly connected layer and is used to forecast category labels in the output layer. The following is the equation for the fully connected layer:

(5) $$\begin{array}{c} y^{l}=w^{l}x^{l-1}+b^{l}\text{,} \end{array}$$

where *y*^*l*^ is the output of the *l* th fully connected layer, *x*^*l*−1^ is the one-dimensional feature vector after flattening, *w*^*l*^ is the weight matrix, and *b*^*l*^ is the bias.

#### 2.1.2. Ensemble Learning

**Figure 3:**
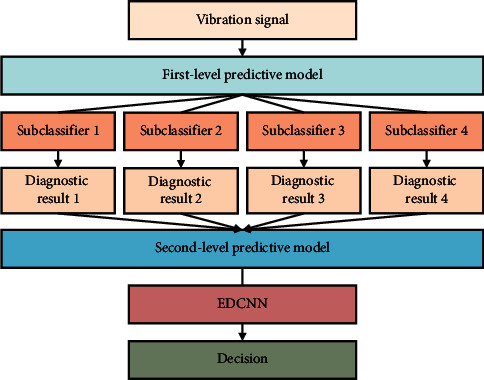
Ensemble learning.

**Figure 4:**
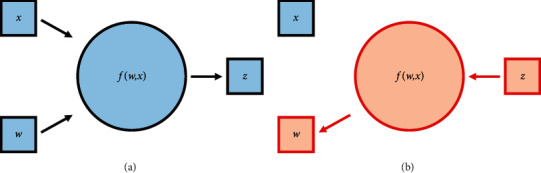
Mechanism of forward and backward propagation. (a) Forward propagation and (b) backward propagation.

Ensemble learning combines several 1D-DCNN subclassifiers into a single prediction model to reduce variance and bias and improve accuracy [32–34]. This study proposes an ensemble 1D-DCNN model approach based on a weighted mechanism as shown in Figure 3. Subclassifiers with different dilation rates initially have the same weights, and the weights are continuously updated based on the outputs of the proposed model. The way of the weighed procedure is shown in the following equation:

(6) $$\begin{array}{c} \hat{y}=argmax\overset{n}{\underset{j=1}{\sum }}\left(w_{j}p_{j}\right)\text{,} \end{array}$$

where *w*_*j*_ is the weights of subclassifiers and *p*_*j*_ is the prediction of subclassifiers, and $\hat{y}$ is the final fault diagnosis decision. Forward and backward propagation mechanisms are present in ensemble model training. Forward propagation is performed by calculating model parameters (subclassifier weights and model weights) and vibration signals to make diagnostic decisions. According to the diagnostic objective, backward propagation finds the most appropriate weights for each neuron and subclassifiers as shown in Figure 4. The cross-entropy loss function [35] and the Adam optimization algorithm [36] play an important role in the backward propagation parameters. The former is a widely used loss function in multiclassification tasks, and the latter effectively minimizes the loss function. With the ensemble learning process, even if a subclassified incorrectly misclassifies faults, associating it with extremely low model weights yields the correct outcome in the final diagnosis of the ensemble model.

#### 2.1.3. Early Stopping Optimization

An optimal diagnostic model with the best generalization performance is generally expected in model training. However, neural network architectures are prone to overfitting. The model may improve as the training and validation subset loss function simultaneously decrease. However, at a certain point in the training process, the loss function of the training subset will continue to decrease while the loss function on the validation subset starts to increase. This is known as overfitting.

**Figure 5:**
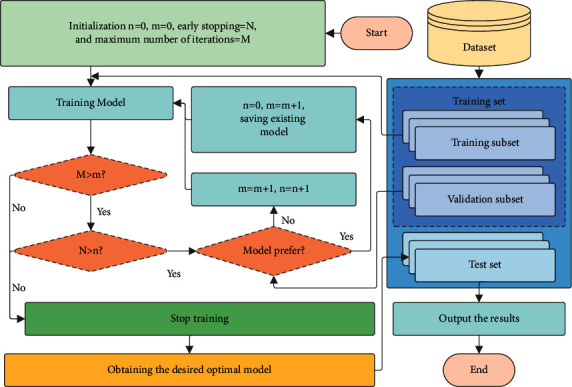
Flowchart of early stopping optimization.

To avoid overfitting, early stopping optimization can be used to stop the model training process depending on model updates as shown in Figure 5. A validating subset loss function-based early stopping optimization is proposed. During each iteration, the model is saved when the loss function of the validation subset decreases. The training process is stopped when the evaluation metric of the model no longer improves, and the number of iterations is within the early stopping optimization. Previous experiments have shown that the results obtained with early stopping do not significantly differ from those obtained with a high number of iterations. However, the computational cost may be several times lower. Early stopping optimization is used in all of the deep learning methods in this research, which is denoted as follows:

(7) $$\begin{array}{c} L_{obt}\left(t\right)=\min_{t^{\prime }<t}L_{va}\left(t^{\prime }\right)\text{,} \end{array}$$

where *t* is the number of iterations, L_obt_(*t*) is the validation subset loss function of the obtained validation subset, and L_va_(*t*′) is the corresponding validation subset loss function at the moment *t*′.

### 2.2. A Detailed Structure of the Intelligent Model

**Figure 6:**
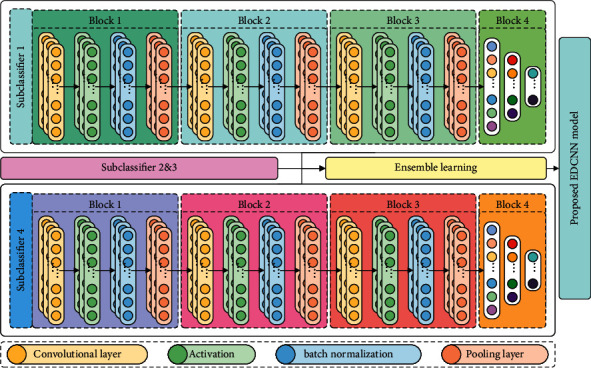
Ensemble dilated convolutional neural network model.

EDCNN consists of a collection of four 1D-DCNN subclassifiers. The structural and parameters of the mathematical model were determined by referring to the paper [37, 38]. Apart from the dilation rate, the hyperparameters of each subclassifier in the proposed model are the same as shown in Figure 6 and listed in Table 1. Blocks 1, 2, and 3 of the subclassifiers serve as feature extractors, while Block 4 serves as the decision maker. Block 1, Block 2, and Block 3 are four-layer dilation CNNs, each containing a dilation convolution layer, a pooling layer, activation, and batch normalization. There are four channels in the first dilation convolution and pooling layer, eight channels in the second dilation convolution and pooling layer, and 16 channels in the third dilation convolution and pooling layer. The convolution kernel has a valid length of 5 with a stride of 1, and the pooling kernel has a valid length of 2 with a stride of 2. Block 4 is a three-layer fully connected neural network with the first layer (input layer) dimension as a flattened input dimension, the second layer (hidden layer) dimension as 128, and the third layer (output layer) dimension as a fault category. In addition, to save model training time and model convergence performance, this study sets the early stop to 5, the maximum number of iterations to 100, the learning rate to 10^−4^, and the small batch size to 100.

### 2.3. Proposed EDCNN-Based Intelligent Fault Diagnosis Scheme

**Figure 7:**
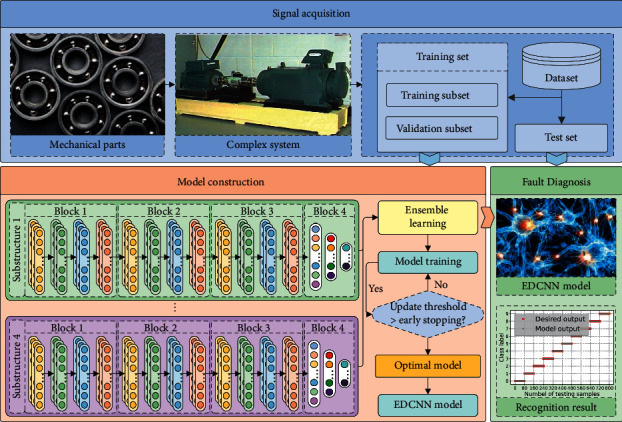
Proposed intelligent fault diagnosis scheme.

A new adaptive deep learning fault diagnosis scheme is proposed based on the advantages of the proposed EDCNN method. The flowchart of this scheme is shown in Figure 7, and the specific steps are as follows:

  Step 1: Signal acquisition. Acceleration sensors are used to collect vibration acceleration signals from rotating machinery and divide them into a training set (which includes a training subset and a validation subset) and a test set.
  Step 2: Model construction. The EDCNN model is built using the training set as the input. The training subset is utilized for initial prediction model training, and the validation subset is used to stop model training at the proper moment in conjunction with early stop optimization.
  Step 3: Fault diagnosis. The testing set is input to the prediction model for achieving end-to-end intelligent fault diagnosis.

## 3. Experimental Study

In this section, to evaluate the advanced diagnostic performance of the proposed fault diagnosis model, several experiments are carried out on Case Western Reserve University (CWRU) [39] rolling bearing and wind turbine dataset. Data acquisition is performed through a sliding time window, where the window size and overlapping structure are 1024 and 128, respectively. The dataset is composed of 800 sets of signals for each fault category. The training and test sets are divided by the dataset by a ratio of 0.8 : 0.2, and the training and validation subsets are divided by the training set by a ratio of 0.8 : 0.2. Moreover, several most advanced methods are selected for comparative analysis. Finally, for this experiment, the deep learning library PyTorch (version 1.9) was utilized, the suggested model was evaluated and implemented in Python (version 3.7), and the experiment was repeated ten times to eliminate random effects.

### 3.1. Comparative Methods

The following diagnosis methods are implemented for comparison to verify the superiority of the proposed model in fault diagnosis (the proposed EDCNN fault diagnosis method is abbreviated as FD-6):

  FD-1: FD-1 is a fault diagnosis method based on the modified support vector machine, which employs the multiscale permutation entropy, linear local tangent space alignment, and least square support vector machine algorithms. According to reference [40], the settings are configured.
  FD-2: FD-2 is a fault diagnosis method based on an artificial neural network. The ANN simulates the structure and function of neural networks in the brain, using mathematical models to model the activity of neurons. In this study, a three-layer ANN was used.
  FD-3: FD-3 is a fault diagnosis method based on an improved recurrent neural network. The improved method, called the gated recurrent unit, can extract time-series features automatically. The training efficiency is significantly higher than that of long short-term memory due to the unique individual gate mechanism.
  FD-4: FD-4 is a fault diagnosis method based on the CNN, which can classify input data according to its hierarchical structure in terms of shifted variables using representational learning. The model used is the subclassified 1 mentioned above.
  FD-5: FD-5 is a fault diagnosis method based on the DCNN. The DCNN has a wider receptive field than the normal convolutional neural network and may capture longer dependencies. The model utilized is subclassifier 2 from the previous section.

### 3.2. Evaluation Metric

**Figure 8:**
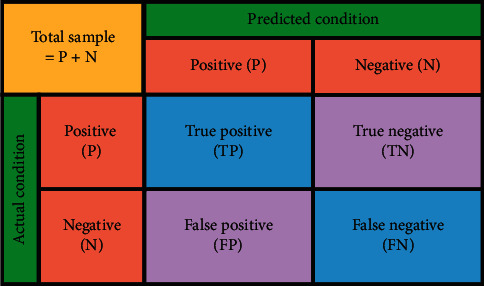
Meaning of TP, TN, FP, and FN.

To quantify the performance of the suggested intelligent fault diagnosis scheme, evaluation metrics were devised. The F score [41], a composite metric that combines precision and recall, is used as the evaluation criterion as follows:

(8) $$\begin{array}{c} \begin{array}{c} Precision=\frac{TP}{TP+FN},\\ Recall=\frac{TP}{TP+FP},\\ F\;score=\frac{\left(1+\beta^{2}\right)*Precision*Recall}{\beta^{2}*Precision+Recall}, \end{array} \end{array}$$

where TP denotes true positive, TN denotes true negative, FP indicates false positive, and FN indicates false negative. Their respective roles are shown in Figure 8; *β*^2^ denotes the weights of precision and recall in the evaluation metrics. Here, *β*^2^ is taken as 1, indicating that equal importance is given to precision and recall. Therefore, it is called the F1 score.

### 3.3. Case Study 1: Experimental Analysis with the CWRU Dataset

#### 3.3.1. Dataset Description

**Figure 9:**
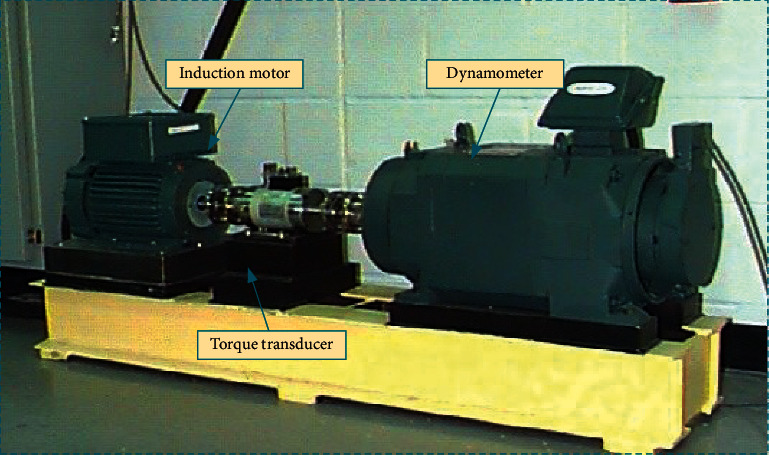
Experimental platform used to obtain the CWRU bearing data.

The CWRU dataset is a remarkable and representative rolling bearing fault diagnostic dataset that has been utilized in many studies to validate condition monitoring and fault diagnosis methods for rotating motors [42, 44]. It is used as a benchmark in this work for experimental investigations to verify the advantages of the proposed EDCNN-based fault diagnosis method. The CWRU experimental platform is shown in Figure 9, which mainly consists of an induction motor, a torque transducer, a dynamometer, and an electronic controller. The vibration signals were collected from a faulty bearing mounted at the end motor fan and sampled at 12 kHz. This dataset studied ten single fault conditions corresponding to standard and different fault diameters for ball faults, inner race faults, and outer race faults as shown in Table 2.

#### 3.3.2. Experimental Validation

**Figure 10:**
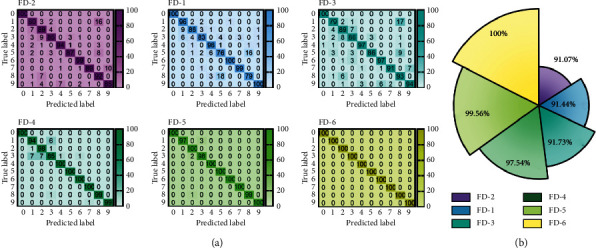
Diagnostic performance of different methods for the CWRU dataset: (a) confusion matrix and (b) the mean F1 scores.

The diagnostic performance in the CWRU experimental platform test set is depicted in Figure 10. In the first set of experiments, only the FD-6 indicated correctly recognized all fault kinds, whereas the remainder of the FD-1, FD-2, FD-3, FD-4, and FD-5 were misdiagnosed, as seen in Figure 10(a). In the repeated experiments, the average F1 scores of each diagnostic model are represented in Figure 10(b). The F1 scores of FD-1, FD-2, and FD-3 are inferior to CNN-based approaches (FD-4, FD-5, and FD-6). The reason for this is that, as compared to FD-1, FD-2, and FD-3, CNN-based fault detection methods have more powerful feature extraction capabilities for identifying various types of faults. Furthermore, the F1 scores of FD-6 are 2.46% and 0.44% higher in the CNN-based fault diagnosis method than those of FD-4 and FD-5, respectively. In comparison to the limited pattern recognition capability of other methods, the suggested FD-6 diagnostic model correctly identifies all health states in the benchmark experiments.

#### 3.3.3. Robustness Analysis

To simulate fault diagnosis scenarios under complex operating scenarios, Gaussian white noise of 4 dB, 2 dB, 0 dB, −2 dB, and −4 dB is added to the original signals, respectively. The comparison approaches and the suggested EDCNN method were tested for robustness in the presence of additional noise.

**Figure 11:**
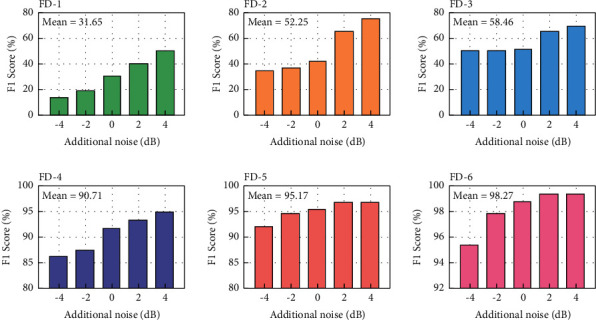
Comparative learning in CWRU tasks with different additional noise levels.

The robustness analysis results of six fault diagnosis methods are shown in Figure 11. It can be concluded that in the presence of additive noise, the classification performance of the diagnostic model deteriorates as the signal-to-noise ratio decreases. The CNN-based fault diagnosis model still outperforms the FD-1, FD-2, and FD-3 diagnostic approaches. The F1 score of FD-6 in the CNN-based diagnostic model is 98.27%, which is better than the F1 scores of FD-4 and FD-5, which are 90.71% and 95.17%, respectively. Furthermore, the presence of a dilated convolution mechanism improves the accuracy and robustness of the fault identification effect. In both sets of CWRU experiments, the suggested FD-6 model obtained the best diagnostic results, ensemble multiple dilated convolutional neural networks, and improved diagnostic performance and robustness under multifeature map comprehensive decision, demonstrating the improved diagnostic performance, and robustness of the proposed model based on the addition of multifeature maps.

### 3.4. Case Study 2: Experimental Analysis with the Wind Turbine Dataset

#### 3.4.1. Dataset Description

**Figure 12:**
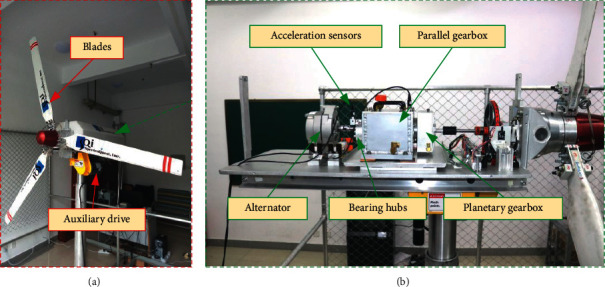
Experimental platform used to obtain wind turbine simulator data.

Wind energy is widely considered to be the most commercially promising and environmentally friendly energy source; however, different harsh working conditions make wind turbines more susceptible to failure. An experimental platform of a wind turbine simulator was created and wind turbine datasets were collected in this work for the aim of wind turbine fault diagnostics. The wind turbine simulation experimental platform consisting of three fan blades, an auxiliary drive, a planetary gearbox, bearing hubs, and an alternator is shown in Figure 12. The vibration signals are sampled from the faulty bearing and the faulty gear phone at one end of the gearbox at 12.8 kHz. Nine health states were investigated using the data collected, including normal, several single fault types, and several compound fault types, as shown in Table 3. Compound faults include mutual interaction between several single fault pulses, degrading diagnostic method identification performance.

#### 3.4.2. Experimental Validation

**Figure 13:**
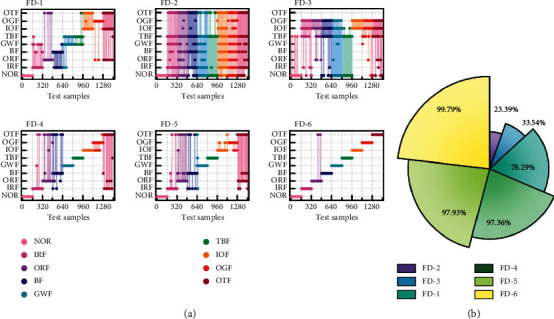
Diagnostic performance of the wind turbine simulation experimental platform: (a) recognition results for a given experiment and (b) the mean F1 scores of different methods.

Compound faults in wind turbines occur concurrently and are coupled by multiple types of faults, posing a significant difficulty for feature extraction, and pattern recognition in fault diagnosis. The fault diagnosis results of the wind turbine experimental platform test set are illustrated in Figure 13. All diagnostic approaches were misdiagnosed in the initial set of wind turbine experiments, as shown in Figure 13(a). The identification results of the remaining approaches demonstrate large-scale misclassification, with the exception of the proposed FD-6 diagnostic method, which misidentifies two fault types. The comprehensive performance of each diagnostic method for repeated experiments on the wind turbine dataset is shown in Figure 13(b). None of the non-CNN-based mathematical models are adequate for diagnosing compound faults. Due to its great feature capability capacity, the suggested FD-6 model is able to retain good recognition performance while dealing with compound fault diagnostic scenarios and is 2.43% and 1.86% ahead of the relatively decent FD-4 and FD-5 in terms of F1 scores.

#### 3.4.3. Robustness Analysis

**Figure 14:**
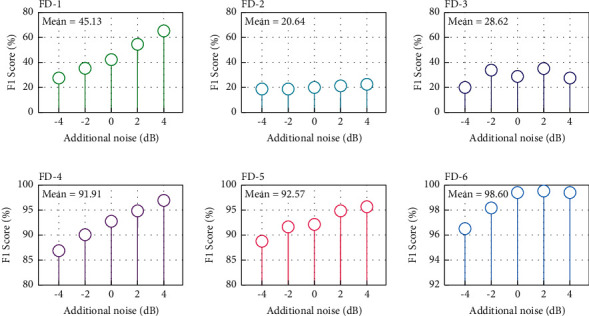
Comparative learning in wind turbine tasks with different additional noise levels.

Likewise, the additional noise was applied to the wind turbine dataset. For different health conditions, 4 dB, 2 dB, 0 dB, −2 dB, and −4 dB additive noise is applied to the vibration signal. The recognition performance of the comparative learning is shown in Figure 14. Obviously, diagnosing compound faults in wind turbines is more difficult than diagnosing single faults in bearings. The performance of the comparison method is still inadequate. Due to CNN's outstanding feature extraction capacity, FD-4, FD-5, and FD-6 outperform FD-1, FD-2, and FD-3. The wider RF of FD-5 and FD-6 feature extractors, which can extract correlation features between longer signals, results in 0.66% and 6.69% better mean performance than FD-4. For the advantage of ensemble learning, FD-6 may gain more effective fault discrimination information in multi-feature maps, resulting in superior classification performance.

## 4. Conclusions

In this paper, an intelligent fault diagnosis approach for rotating machinery is proposed using ensemble dilated convolutional neural networks (EDCNN). On the CWRU bearing dataset and the wind turbine dataset, the proposed approach is examined and validated. The following conclusions can be drawn:

1. In both the bearing and wind turbine datasets, the proposed EDCNN adaptive fault diagnostic approach accurately identifies all single and compound faults.
2. In comparison to advanced fault diagnosis methods (such as MSVM, ANN, GRU, CNN, and DCNN), the suggested EDCNN method can identify all health states correctly and reliably.
3. The robustness analysis results indicate that the suggested EDCNN approach can perform fault diagnosis of rotating equipment in complex situations with stronger feature learning and feature extraction capabilities.

## Figures and Tables

**Table 1:** Parameters of the dilated convolutional neural network model.

Object			Hyperparameter settings
*Feature extractor*	Block 1	Convolutional layer #1	Number of channels: 4, kernel width: 5, stride: 2
		Pooling layer #1	Kernel width: 2, stride: 2
	Block 2	Convolutional layer #2	Number of channels: 8, kernel width: 5, stride: 2
		Pooling layer #2	Kernel width: 2, stride: 2
	Block 3	Convolutional layer #3	Number of channels: 16, kernel width: 5, stride: 2
		Pooling layer #3	Kernel width: 2, stride: 2

*Decision maker*	Block 4	Fully connected layer #1	Network width: input dimension
		Fully connected layer #2	Network width: 128
		Fully connected layer #3	Network width: number of fault category

Early stopping			5
Maximum number of iterations			100
Learning rate			10^−4^
Small batch size			100

**Table 2:** Description of ten working states of the CWRU experimental platform.

Status	Fault diameter	Abbreviation	Fault type	Label	Sample length	Dataset (training subset/validation subset/test set)
Normal	—	NOR	—	0	1024	512/128/160

Ball fault	0.007	B007	Single	1	1024	512/128/160
	0.014	B014	Single	2	1024	512/128/160
	0.021	B021	Single	3	1024	512/128/160

Inner race fault	0.007	IR007	Single	4	1024	512/128/160
	0.014	IR014	Single	5	1024	512/128/160
	0.021	IR021	Single	6	1024	512/128/160

Outer race fault	0.007	OR007	Single	7	1024	512/128/160
	0.014	OR014	Single	8	1024	512/128/160
	0.021	OR021	Single	9	1024	512/128/160

**Table 3:** Description of nine working states of the wind turbine simulator experimental platform.

Status	Abbreviation	Fault type	Label	Sample length	Dataset (training subset/validation subset/test set)
Normal	NOR	—	0	1024	512/128/160
Inner race fault	IRF	Single	1	1024	512/128/160
Outer race fault	ORF	Single	2	1024	512/128/160
Ball fault	BF	Single	3	1024	512/128/160
Gear wear fault	GWF	Single	4	1024	512/128/160
Tooth broken fault	TBF	Single	5	1024	512/128/160
Inner race outer race fault	IOF	Compound	6	1024	512/128/160
Outer race gear wear fault	OGF	Compound	7	1024	512/128/160
Outer race tooth broke fault	OTF	Compound	8	1024	512/128/160

## Data Availability

The data used to support the findings of this study are available from the corresponding author upon request.
